# Neurological update: COVID-19

**DOI:** 10.1007/s00415-021-10581-y

**Published:** 2021-04-30

**Authors:** A. L. Ren, R. J. Digby, E. J. Needham

**Affiliations:** 1 Division of Anaesthesia, University of Cambridge, Cambridge, UK; 2 Department of Clinical Neurosciences, University of Cambridge, Cambridge, UK

**Keywords:** COVID-19, Neuroimmunology, Cerebrovascular disease, Critical illness

## Abstract

Coronavirus Disease 2019 is predominantly a disorder of the respiratory system, but neurological complications have been recognised since early in the pandemic. The major pathophysiological processes leading to neurological damage in COVID-19 are cerebrovascular disease, immunologically mediated neurological disorders and the detrimental effects of critical illness on the nervous system. It is still unclear whether direct invasion of the nervous system by the Severe Acute Respiratory Syndrome Coronavirus 2 occurs; given the vast numbers of people infected at this point, this uncertainty suggests that nervous system infection is unlikely to represent a significant issue if it occurs at all. In this review, we explore what has been learnt about the neurological complications of COVID-19 over the course of the pandemic, and by which mechanisms these complications most commonly occur.

## Introduction

Coronavirus Disease 2019 (COVID-19), the clinical disorder caused by infection with severe acute respiratory syndrome coronavirus 2 (SARS-CoV-2), is characterised predominantly by pneumonitis with subsequent severe hypoxaemic respiratory failure [1]. In addition to the respiratory manifestations, multiple organ dysfunction such as acute kidney injury and myocarditis can occur [2]; only a few months into the pandemic, attention was drawn to the occurrence of neurological symptoms occurring in patients with COVID-19 [3]. Whilst many of these were simply symptoms commonly seen in febrile illnesses, such as headache and myalgia, a vast breadth of neurological disorders resulting from COVID-19 began to appear in the literature, including prolonged disorders of consciousness, immunologically mediated neurological diseases and cerebrovascular insults. At a group-wise level, patients with COVID-19 display elevated levels of brain-injury biomarkers such as glial fibrillary acidic protein (GFAP) and neurofilament light chain (NfL) in a severity-dependent manner, both in the presence and absence of neurological symptoms, highlighting that structural neurological damage occurs even in comparatively mild disease [4–6].

As the pandemic progressed, and clinical trials resulted, it became apparent that a substantial burden of the morbidity caused by the disease comes not from the virus itself, but rather the host’s response to the infection, with immunomodulatory interventions showing substantial survival benefit compared with modest effects from the use of antiviral agents [7–9]. In a similar vein, convincing reports of encephalitis caused by direct SARS-CoV-2 infection remain scant given the millions of individuals who have been infected, suggesting that the neurological sequelae of COVID-19 do not result from direct infection per se, but rather from indirect consequences such as inflammation and hypercoagulability. Knowing to what degree these complications of infection are specific to COVID-19, or even more common in COVID-19 than in other infectious diseases, remains an unresolved issue. In many regards, it would preferable if COVID-19 was not unique, as the transferrable lessons learnt from the unprecedented research effort undertaken during the pandemic may be one positive outcome from what has otherwise been a bleak period in human history.

**Fig. 1:**
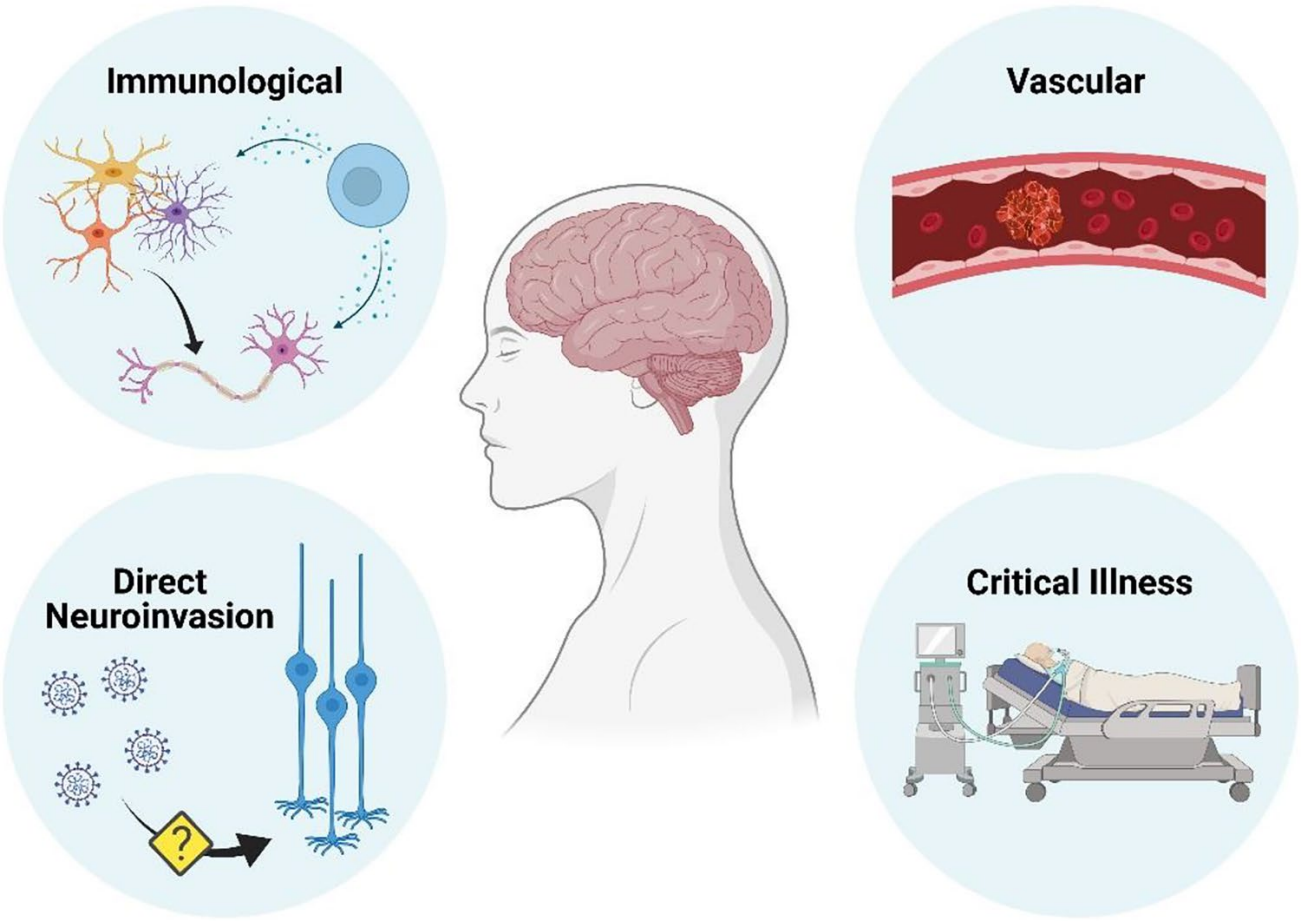
Mechanisms by which neurological disease can occur as a result of COVID-19. Vascular disease appears to be disproportionately common in COVID-19 than in comparable infections, whereas immunologically mediated neurological conditions are similar in frequency. The evidence supporting direct central nervous system by SARS-CoV-2 as a cause of neurological disease is scant. *Figure created with BioRender.com*

In this narrative review, we summarise what has been learnt about the acute neurological complications of COVID-19, and discuss the four main proposed pathophysiological processes which lead to their occurrence (Fig. 1). The chronic neurological and neuropsychiatric consequences of COVID-19, including both the long-term sequelae of acute neurological disease, and the less well-defined syndrome of “Long-COVID” [10], are likely to have significant public health implications, but are beyond the scope of this review.

## Direct central nervous system invasion by SARS-CoV-2

In the landmark paper which first highlighted the neurological system as a potential target of COVID-19, Mao et al. concluded (without corroborating data) that the occurrence of neurological symptoms in these patients was evidence that SARS-CoV-2 could infect the nervous system [3]. Whilst no data existed to support this assertion directly at this point of the pandemic, SARS-CoV (the causative agent of the 2003 SARS outbreak) had previously been isolated from brain tissue in human autopsies, thus suggesting the potential for coronaviruses to invade the central nervous system (CNS) [11], and in light of the extensive genetic similarities between SARS-CoV and SARS-CoV-2 [12], it was reasonably hypothesized that SARS-CoV-2 may also have neuroinvasive capabilities [13].

An important factor determining the likelihood of direct cerebral invasion by SARS-CoV-2 is the expression within the CNS of the angiotensin converting enzyme 2 (ACE2) receptor, a key mediator for SARS-CoV-2 entry into multiple cell types [14]. A study using human iPSC-derived brain organoids and a transgenic mouse model demonstrated the necessity of ACE2 expression for neuroinvasion, finding that treatment of organoids with anti-ACE2 monoclonal antibodies prevented SARS-CoV-2 invasion of neurons, and intranasal inoculation of SARS-CoV-2 in mice overexpressing human ACE2 resulted in widespread viral invasion in the brain [15]. Given the extremely low expression of ACE2 in human brains and olfactory sensory neurons [16, 17], we would interpret from these results that the risk of direct brain infection is likely to be very small, although it is feasible that ACE2 expression could be upregulated by certain disease states [18], thereby facilitating entry in particular situations. In addition, other cell surface receptors are being investigated for their ability to potentiate ACE2-mediated SARS-CoV-2 cell entry. One prominent example is neuropilin-1 (NRP1), which is highly expressed in the olfactory epithelium and has been shown to facilitate SARS-CoV-2 infectivity in vitro [19, 20]; NRP1 is expressed ubiquitously throughout the body, and unlike ACE2 is well-expressed in the central nervous system [21], and so could represent a more tractable mechanism of direct neurological infection, although this has yet to be demonstrated.

The current evidence for direct viral invasion of neurons occurring in human patients is conflicting, with certain groups describing frequent viral presence [22–25], but many others finding no evidence of it at all [23, 26–29], suggesting a substantial impact of technical approach. Importantly, no descriptions of viral inclusions or reactive cellular changes typical of true infection have been reported [30]. Such reports have largely used reverse transcription PCR on brain tissue for viral detection, but the potential for contamination from blood or endothelium using this technique is high; visualisation of virus with immunohistochemistry is not only far less commonly reported [22, 30–32], but also presents difficulties in interpretation, as noted by Solomon et al. [31]. Of note is the distinct lack of inflammatory response at sites where viral particles were visualised; some authors have suggested that this may indicate an evasion of the immune-system, whereas our suggestion is that these particles are likely to be contaminants and not indicative of neuronal infection per se.

If SARS-CoV-2 is to reach the CNS, the virus must be able to circumvent the blood brain barrier (BBB) [33]. Frequently debated mechanisms of CNS entry include transcribial migration from olfactory sensory neurons [34], retrograde transsynaptic transmission starting from peripheral nerves [13], and hematogenous spread from the circulatory system [35].

Anosmia and ageusia are common symptoms of COVID-19 [36], and the olfactory epithelium are some of the earliest cell types to be infected [37]. Theoretically, if the virus can pass to olfactory sensory neurons, it could travel across the cribriform plate to arrive at the olfactory bulb, and subsequently access other brain regions [34]. However, this same mechanism would be open to all respiratory viruses, and it is clear that cerebral invasion, whilst it can occur with infections such as influenza, is extremely rare [38].

Similarly, should SARS-CoV-2 be able to infect other cranial or peripheral nerves, it would introduce the possibility of retrograde viral transport up the peripheral axon followed by transsynaptic migration to a neighboring neuron. Through this mechanism, a virus could hop across a series of synaptically connected neurons to ultimately arrive at neurons in the brain and spinal cord [39]. Multiple viral strains, such as herpes simplex virus-1 and rabies virus, exhibit a retrograde CNS infection strategy [40]; however, whilst trigeminal, vagus, and enteric neurons have been proposed as entry points for SARS-CoV-2 CNS infection based on the presence of certain symptomatologies [41], there is no direct evidence of peripheral nerve infection, let alone as a precursor to CNS neuroinvasion.

Whereas the prior two hypothesized routes for CNS entry require infection of peripheral or cranial nerves, the haematogenous route entails either transcytosis across the vascular endothelium of the BBB, or a Trojan horse-like reliance on infected host immune cells to bypass the BBB [42–44]. ACE2 is highly expressed on arterial and venous endothelial cells [14], and endothelial invasion by virus is well described [45], as is leukocyte infection [43], but neither of these explanations make the final leap to explaining passage into neurons themselves.

## Immunologically mediated neurological disorders

Aberrant immunological responses to infection are a well recognised cause of both peripheral and central nervous system disease, and are broadly categorised as either parainfectious (the result of bystander damage from innate immune responses occurring at the time of acute infection) or post-infectious (erroneous adaptive autoimmune responses typically occurring in the weeks following acute infection) disorders. The most common of these conditions is Guillain-Barré syndrome (GBS), a post-infectious neuropathy which is thought to occur as a result of molecular mimicry between epitopes on the pathogen and peripheral nerve structures. There have now been numerous reports and case-series of GBS and related disorders such as Miller-Fisher Syndrome occurring following COVID-19 [46–51], but unlike in the Zika virus pandemic, there does not seem to be a disproportionately high risk of developing GBS following COVID-19 compared with other viruses. Indeed, in a large United Kingdom paired epidemiological and cohort study, the incidence of GBS in the UK was lower during the pandemic than in previous years, presumably because of reduced transmission of higher risk pathogens such as campylobacter jejuni [52]. Whilst no definite evidence of molecular mimicry has been demonstrated to explain the occurrence of GBS following COVID, comparison of SARS-CoV-2 sequencing data with primary amino acid sequences of human proteins known to be associated with immune-mediated neuropathies identified two immunologically relevant hexapeptides which were present in both datasets [53], raising the possibility of this mechanism.

The rarer, but immunologically related demyelinating central nervous system diseases of acute disseminated encephalomyelitis and transverse myelitis have also been reported following COVID-19 [54–58], but again the relatively scarce reports in the literature would suggest that these phenomena are not happening disproportionately frequently in patients with COVID-19.

With regards to parainfectious immunological diseases of the nervous system, a case of acute necrotising encephalopathy was reported early in the pandemic [59]. This is a rare condition characterised by symmetrical necrotising lesions in the thalami which usually occurs in children (often, but not invariably with a mutation in the RANBP2 gene) in the context of influenza infection, and is thought to be the result of a dysregulated cytokine response in conjunction with blood–brain barrier breakdown [59, 60]. Given the contemporaneous interest in the role of the “cytokine storm” (an excessive production of proinflammatory cytokines leading to widespread tissue damage and subsequent multiple organ failure) as a potentially important component of COVID-19 morbidity [61], acute necrotising encephalopathy stood to reflect an extension of the cytokine-driven systemic illness into the CNS, but comparatively few further cases have been forthcoming given the total number of infections [50, 59, 62–66]. Whilst true acute necrotising encephalopathy is rare, encephalopathy more broadly is common in COVID-19, and ubiquitous in very severe disease [67]. A role of a hyperinflammatory state and the “cytokine storm” in the pathogenesis of this encephalopathy has been suggested, and homology with Immune effector cell-associated neurotoxicity syndrome (ICANS) and cytokine-release syndrome (CRS) seen after Chimeric Antigen Receptor (CAR) T-cell therapy has been drawn [68, 69]. Marked upregulation of systemic inflammation induces permeability of the blood brain barrier [70, 71], with subsequent microglial and astrocytic activation which sets in motion a positive feedback loop of local inflammation [72–74]; this local inflammation, in conjunction with a multitude of other contributory factors, including neurotransmitter imbalances, brain energy metabolism dysfunction and the deleterious effects of medications and toxic levels of endogenously produced compounds such as urea, compromises brain function leading to encephalopathy [74].

A number of neuropathological studies of COVID-19 patients, even without a distinct neurological syndrome, have demonstrated CNS inflammatory changes, with microglial activation, astrocytosis and perivascular T-cell cuffing, most commonly in the medulla oblongata [22, 28, 75], however, similar changes have been described in other severe inflammatory states [76, 77], and it is unclear whether these changes have any specificity to COVID-19, or are simply indicative of severe infectious disease.

A host of other individual cases of autoimmune neurological diseases (such as myasthenia gravis, neuromyelitis optica spectrum disorder and antibody-mediated encephalitis) occurring in the context of COVID-19 have been published [78–81], but whether these represent coincidental disease processes (given the ubiquity of COVID-19), or the common result of an immune dyscrasia, such as the lymphopoenia often seen in COVID-19 [82, 83], is not established.

## Neurovascular and thromboembolic disease

The relationship between vascular disease and COVID-19 appears to be complex; from early in the pandemic it became clear that venous thrombosis was a common occurrence [84], and that even in the absence of overt venous thromboembolism, D-dimer levels were markedly raised [85], suggesting activation of the coagulation factors in the microvasculature.

Regarding macrovascular neurological disease, it appears that COVID-19 may increase the risk of stroke greater than influenza infection [86]. Particularly, there appears to be a predilection towards large vessel occlusions, with some series even describing a reduction in the occurrence of small vessel infarction [87], although the possibility that patients with milder strokes did not seek medical attention given the health-system situation at the time should not be underestimated as a cause for this shift in pattern of disease [88]. The exact mechanism by which COVID-19 increases the risk of stroke is uncertain; there is a disproportionately high rate of cryptogenic stroke [89–91], i.e. not explained by large vessel atherosclerosis or cardioembolism, suggesting that the increase is not simply due to a decompensation of these processes. In those with severe respiratory involvement, and subsequently high right-heart pressures, right to left shunting of venous thromboemboli via patent foramen ovale is possible [92], but would not explain the incidence in lower severity COVID-19. More likely would be the contributions of both coagulopathy and endotheliopathy. Whilst severe illness is well known to predispose to hypercoagulability via a number of mechanisms such as immobility and dehydration, COVID-19 appears to disproportionately increase thrombotic tendency [85], and often patients display a frank coagulopathy on routine laboratory testing, with prolonged prothrombin and activated partial thromboplastin times, raised fibrinogen degradation products and d-dimer [93]. Furthermore, the occurrence of immunologically mediated thrombosis has been mooted by the comparatively frequent presence of lupus anticoagulant, as well as of anticardiolipin and antiphospholipid antibodies in patients with COVID-19 [94, 95], although the relevance of these autoantibodies in this setting is not clear.

In addition to the “haematological” predisposition toward thrombosis, endothelial disruption appears to be of particular importance in COVID-19, and would further contribute to the risk of thrombosis and subsequent stroke [96]. Post-mortem studies have demonstrated viral inclusions within endothelial cells (which richly express the ACE2 receptor [97]), with a significant associated inflammatory response leading to endotheliitis detectable in many organs including lung, heart, kidney and intestine, far in excess than the comparable condition of influenza [45, 98, 99].

Whilst these disorders of coagulation and endothelial integrity represent potential drivers of large vessel cerebrovascular disease, they also raise the possibility that impairment of the microvasculature might be involved in some of the less clear-cut neurological presentations, such as prolonged disorders of consciousness (including akinetic-mutism states) [63, 100–102], myoclonus (a relatively common complication with over 50 cases reported in the literature**,** which is seemingly unrelated to hypoxic ischaemic brain injury [103]), or even on the long-term cognitive outcomes of those who recover. Indeed, neuroimaging studies have commonly described cerebral microhaemorrhages occurring in those with severe COVID-19, reminiscent of microangiopathic disorders such as amyloid and hypertensive angiopathies [100, 104, 105], a phenomenon borne out in pathology series [32, 75, 100, 106]. As would be expected in a disorder of the endothelium, similar microvascular changes have been described in multiple organ systems [107], and it has even been suggested that this process might be the cause of the mononeuritis multiplex seen in a substantial minority of survivors of severe COVID-19 [108].

It should be noted that similar cerebral microvascular processes have been described in other relevant disease states, such as hypoxic ischaemic brain injury and acute respiratory distress syndrome from other causes [109, 110]**.** Whilst studies undertaking direct contemporaneous comparison between COVID-19 and similar conditions are extremely rare, there is a suggestion that systemic vascular complications are more common in COVID-19 [86, 99, 111], and of all the neurological complications of COVID-19, it may well be this aspect which is the most pathognomonic.

## Neurological sequelae of critical illness

Aside from those aspects of COVID-19 which may specifically predispose to neurological disease, a substantial burden of disability will occur simply by way of the prolonged intensive care treatment experienced by so many patients. Hypoxic-ischaemic encephalopathy resulting from persistent hypoxaemia will undoubtedly leave a legacy of neurological impairment, and yet the literature has yet to delineate the prevalence of this. Delirium is commonplace in critically ill patients [112], and will have been exacerbated during the pandemic with the necessary use of older sedation agents given medication shortages [113]; the occurrence of delirium is well recognised to have detrimental long-term consequences on cognition [114]. Whilst the true incidence of encephalopathy in COVID-19 has not been fully delineated, it appears in the majority of patients requiring ICU admission [67], and EEG studies suggest that is likely to represent one of the most common acute neurological disturbances, with altered mental status being the most frequent indication for an EEG request, and diffuse slowing being the most frequently detected abnormality [115]. Interestingly, there appears to be a predominance of frontal lobe EEG abnormalities in COVID-19, which has been raised as a potentially pathognomonic feature of encephalopathy in COVID-19; other described abnormalities, such as epileptiform discharges, are commonly the result of underlying or pre-existing neurological pathology [115]. Prolonged periods of immobility, and particularly the use of prone-positioning for the amelioration of refractory hypoxaemia are associated with compressive and traction neuropathies and plexopathies [50, 116, 117]. Common risk factors for the development of critical illness polyneuromyopathy, such a prolonged ventilation, systemic inflammatory response syndrome, acute respiratory distress syndrome and multiorgan failure [118] are all frequently seen in COVID-19 [63], leading to a high occurrence of this condition [119]. Lastly, ECMO, the salvage therapy for those who can not be adequately oxygenated with invasive mechanical ventilation, is associated both with intracranial haemorrhage (largely as a result of the necessary anticoagulation) and ischaemic stroke [120, 121].

## Conclusions

The COVID-19 pandemic has resulted in a substantial burden of neurological disease through various mechanisms, most notably through a preponderance towards vasculopathy and thromboembolism, the consequences of critical illness and immunological diseases triggered by infection. Whilst direct comparison with other infectious disease is challenging, it is not apparent that autoimmune complications are more common than following other similar infections, but there is a suggestion that the vascular and thrombotic manifestations are more pronounced in COVID-19 than in similar infections. Direct central nervous system infection by SARS-CoV-2 does not appear to occur with any significant frequency, if indeed it happens at all. Whilst hopefully an event which will not recur in our lifetimes, the COVID-19 pandemic has provided an unparalleled opportunity to study the consequences of severe illness on the nervous system, and it is our hope that in time the lessons learnt from this crisis may stand to improve the management and subsequent neurological health of critically unwell patients more broadly.
